# Sarcopenia is independently associated with poor preoperative physical fitness in patients undergoing colorectal cancer surgery

**DOI:** 10.1002/jcsm.13536

**Published:** 2024-06-26

**Authors:** Jason Rai, Edward T. Pring, Katrina Knight, Henry Tilney, Judy Gudgeon, Mark Gudgeon, Fiona Taylor, Laura E. Gould, Joel Wong, Stefano Andreani, Dinh V.C. Mai, Ioanna Drami, Phillip Lung, Thanos Athanasiou, Campbell Roxburgh, John T. Jenkins

**Affiliations:** ^1^ BiCyCLE Research Group St Mark's the National Bowel Hospital London UK; ^2^ Department of Surgery and Cancer Imperial College London London UK; ^3^ Department of Surgery, School of Medicine, Dentistry & Nursing University of Glasgow Glasgow UK; ^4^ Frimley Park Hospital Frimley Health NHS Foundation Trust Frimley UK; ^5^ Whipps Cross University Hospital Barts Health NHS Trust London UK

**Keywords:** Body composition, Cardiopulmonary exercise testing, Colorectal cancer surgery, Myosteatosis, Sarcopenia, Visceral obesity

## Abstract

**Background:**

Accurate preoperative risk assessment for major colorectal cancer (CRC) surgery remains challenging. Body composition (BC) and cardiopulmonary exercise testing (CPET) can be used to evaluate risk. The relationship between BC and CPET in patients undergoing curative CRC surgery is unclear.

**Methods:**

Consecutive patients undergoing CPET prior to CRC surgery between 2010 and 2020 were identified between two different UK hospitals. Body composition phenotypes such as sarcopenia, myosteatosis, and visceral obesity were defined using widely accepted thresholds using preoperative single axial slice CT image at L3 vertebrae. Relationships between clinicopathological, BC, and CPET variables were investigated using linear regression analysis.

**Results:**

Two hundred eighteen patients with stage I–III CRC were included. The prevalence of sarcopenia, myosteatosis, and visceral obesity was 62%, 33%, and 64%, respectively. The median oxygen uptake at anaerobic threshold (VO2 at AT) was 12.2 mL/kg/min (IQR 10.6–14.2), and oxygen uptake at peak exercise (VO2 peak) was 18.8 mL/kg/min (IQR 15.4–23). On univariate linear regression analysis, male sex (*P* < 0.001) was positively associated with VO2 at AT. While ASA grade (*P* < 0.001) and BMI (*P* = 0.007) were negatively associated with VO2 at AT, on multivariate linear regression analysis, these variables remained significant (*P* < 0.05). On univariate linear regression analysis, male sex (*P* < 0.001) was positively associated with VO2 peak, whereas age (*P* < 0.001), ASA grade (*P* < 0.001), BMI (*P* = 0.003), sarcopenia (*P* = 0.015), and myosteatosis (*P* < 0.001) were negatively associated with VO2 peak. On multivariate linear regression analysis age (*P* < 0.001), ASA grade (*P* < 0.001), BMI (*P* < 0.001), and sarcopenia (*P* = 0.006) were independently and negatively associated with VO2 peak.

**Conclusions:**

The novel finding that sarcopenia is independently associated with reduced VO2 peak performance in CPET supports the supposition that reduced muscle mass relates to poor physical function in CRC patients. Further work should be undertaken to assess whether sarcopenia diagnosed on CT can act as suitable surrogate for CPET to further enhance personalized risk stratification.

## Introduction

In an age attempting to personalize medicine, preoperative risk assessment for major colorectal cancer (CRC) surgery remains a challenge. Body composition analysis and objective physical fitness assessment before surgery can be used to evaluate risk.^1^, ^2^, ^3^ Accurate identification of the high‐risk patient, predicted to have poor outcome, can help facilitate shared decision making, direct prehabilitation, optimize co‐morbidities, and guide intra‐operative and post‐operative care.

Body composition metrics can be derived from routine preoperative computed tomography (CT) scans of CRC patients.^4^, ^5^ A single slice axial CT image at the level of the third lumbar vertebra (L3) can be used to estimate body composition, and it has been proven as a relevant clinical biomarker.^6^, ^7^, ^8^ Sarcopenia, reduced muscle mass and strength, has been associated with increased postoperative morbidity and poorer prognosis in CRC.^9^, ^10^ Myosteatosis, the deposition of ectopic fat within skeletal muscle, is independently associated with poorer overall survival in multiple primary cancers, including CRC.^11^, ^12^, ^13^, ^14^ Visceral obesity, abnormally high deposition of adipose tissue in the intra‐abdominal cavity, has been associated with longer hospital stay, higher morbidity, and worse disease free and overall survival in CRC.^4^, ^15^, ^16^

Cardiopulmonary exercise testing (CPET) provides an objective assessment of physical fitness through evaluation of pulmonary, cardiovascular, haematopoietic, neuropsychological, and skeletal muscle systems that are not reflected by measurement of an individual organ system functions.^3^, ^17^ CPET involves measurements of respiratory oxygen uptake (VO2), carbon dioxide production (VCO2), and ventilatory measures during symptom limited exercise. CPET can be used as a preoperative test to objectively evaluate perioperative risk and identify patients at risk of adverse outcome.^2^, ^3^ However, in clinical practice, CPET is not routinely available while CRC patients constantly undergo staging CT scan providing opportunistic body composition analysis. The role of CT‐derived body composition as a preoperative physical fitness tool deserves further research.

Emerging evidence suggests that myosteatosis is associated with reduced physical fitness when objectively evaluated by CPET in cancer patients undergoing hepatopancreatobiliary surgery.^18^ Interestingly, in oesophago‐gastric cancer patients undergoing resection, chest wall skeletal muscle mass was associated on CPET with oxygen uptake at anaerobic threshold (VO2 at AT) and at peak exercise (VO2 peak), rather than myosteatosis.^19^ The relationship between body composition and CPET in CRC patients remains unclear. The aim of this study is to investigate the association between preoperative CT body composition phenotype and CPET variables in colorectal cancer patients undergoing curative surgery.

## Methods

The study was reviewed and approved by the Queens Square Research Ethics Committee (20/LO/0370), NHS Health Research Authority, London. Due to the retrospective nature of the study, the need for written informed consent was waived. CPET was performed in two colorectal cancer units: Frimley Park Hospital (Surrey, UK) and Whipps Cross University Hospital (London, UK). Retrospective analysis of prospective maintained data was undertaken at St Mark's Hospital (London, UK) and Glasgow Royal Infirmary (Glasgow, UK). CT body composition analysis was carried out at St Mark's Hospital.

Patients undergoing CPET prior to colorectal resection surgery with curative intent were identified between 2010 and 2020. All patients had a histologically confirmed diagnosis of colorectal adenocarcinoma. The inclusion criteria for CPET at Whipps Cross University Hospital included all patients undergoing elective colorectal resection surgery from 2018 to 2020 as the CPET service was established from 2018. CPET was considered for all patients undergoing elective rectal resection surgery at Frimley Park Hospital; however, it was not performed on patients undergoing colon resection. High anaesthetic risk patients with past medical history of severe aortic stenosis, recent myocardial infraction or unstable angina and severe heart failure were assessed by the anaesthetic team and when appropriate excluded from CPET. Limitation to perform task on CPET cycle ergometer due to limb surgery or amputation excluded patients from CPET. Patients who underwent emergency, palliative, local (organ preserving) resection, and those without preoperative CT scans were excluded.

## Computed tomography body composition analysis

Each patient's preoperative staging CT scan performed nearest and prior to the date of surgery was selected for body composition analysis. Single axial slice CT image at L3 was extracted from the picture archiving and communication system (PACS) in digital imaging and communications in medicine (DICOM) format. CT images were analysed using Data Analysis Facilitation Suite (DAFS) version 3.7 (Voronoi Health Analytics, Vancouver, Canada). Cross‐sectional areas were automatically segmented using predefined Hounsfield unit (HU) ranges for skeletal muscle (SM) (−29 to 150 HU), visceral adipose tissue (VAT) (−150 to −50 HU), and subcutaneous adipose tissue (SAT) (−190 to −30 HU). Tissue segmentation was further manually verified by a trained clinician. The cross‐sectional muscle area (cm^2^) was normalized by height squared to calculate the lumbar skeletal muscle index (cm^2^/m^2^) (SMI).

## Body composition phenotype classification

Sarcopenia was defined according to patient sex and body mass index (BMI) using accepted values.^20^ Sarcopenia was defined as SMI < 43 cm^2^/m^2^ in men with BMI < 25. In men with BMI ≥ 25, sarcopenia was defined as SMI < 53 cm^2^/m^2^. Sarcopenia in female was defined as SMI < 41 cm^2^/m^2^. Myosteatosis was defined according to patient BMI using skeletal muscle mean attenuation (SM‐MA).^20^ Myosteatosis in patients with BMI < 25 was defined as SM‐MA < 41 HU. In patients with BMI ≥ 25, myosteatosis was defined as <33 HU. Visceral obesity was defined as visceral fat area >164 cm^2^ in men and >80 cm^2^ in female.^21^

## Cardiopulmonary exercise testing

CPET was performed according to standardized methods published by Perioperative Exercise Testing and Training Society and endorsed by the association for respiratory technology & physiology in the United Kingdom.^22^ Heart rate, peripheral oxygen saturation, non‐invasive blood pressure, and 12 lead ECG traces were monitored. Test procedure included cycle ergometer with 3 min at rest and 3 min unloaded cycling followed by an incremental ramp protocol. The supervising clinician or physiologist encouraged the patient to exercise to maximal capacity. The patient determined the cessation of the test. CPET was terminated with a minimum recovery time of 3 min during which the patient continued to pedal at a self‐determined pace against no resistance. CPET variables measured were oxygen uptake at anaerobic threshold (VO2 at AT), oxygen uptake at peak exercise (VO2 peak), ventilatory efficiency, and oxygen pulse. CPET results were interpreted and reported by trained staff with recognized clinical experience.

Clinical and pathological data including perioperative complications were recorded retrospectively. Pathological tumour stage was recorded using the TNM staging system.^23^ Post‐operative complications were classified using Clavien–Dindo classification (I to V).^24^ Anastomotic leak and 30 day mortality were also recorded.

## Statistical analysis

Statistical analysis was performed using SPSS software version 29.0.1.0 (SPSS Inc., IBM, Chicago, Illinois, USA). Descriptive statistics were used to summarize baseline characteristics. Continuous variables were presented as median, interquartile range (IQR), and compared using an independent *t*‐test or the Mann–Whitney *U* test. Categorical variables were compared using chi‐square or Fisher's exact test.

Relationships between clinico‐pathological, body composition phenotypes, and CPET variables were investigated using linear regression. Variables with a *P*‐value <0.05 on univariate linear regression analysis were entered into the multivariate linear regression model.

## Results

**Figure 1:**
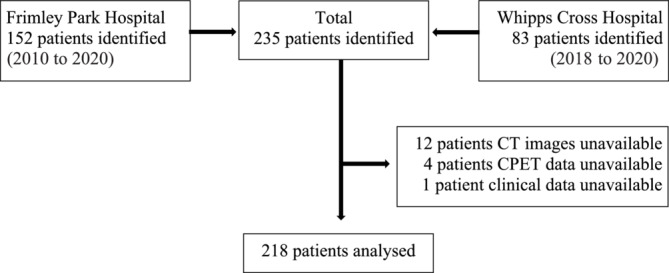
Patient flow diagram.

A total of 218 patients were included in the final analysis. The patient flow diagram is outlined in Figure 1. The baseline demographic and clinico‐pathological characteristics are presented in Table 1.

**Table 1:** Baseline demographic and clinico‐pathological characteristics (*n* = 218)

Variables	*n*	%
Age		
Median (IQR)	67 (60–77)	
Gender		
Female	65	30
Male	153	70
ASA grade		
I–II	156	72
III–IV	62	28
BMI		
Median (IQR)	27 (25–30)	
Tumour site		
Colon	58	27
Rectum	160	73
Pathological tumour (pT) stage		
0–2	73	34
3–4	145	66
Pathological lymph node (pN) stage		
0	126	58
1–2	92	42
Tumour differentiation		
Well to moderate	186	91
Poor	18	9
UICC stage		
I–II	103	47
III	115	53
Surgical approach		
Laparoscopic or robotic	188	86
Open	30	14
Neoadjuvant treatment		
No	178	83
Yes	36	17
Adjuvant treatment		
No	151	69
Yes	67	31

Most patients were male (70%) with median age of 67 (IQR 60–77). The majority of patients underwent surgery for rectal cancer (73%), and most were classified as ASA grade I or II (72%). Laparoscopic or robotic surgery (86%) was mostly performed. All complications (Clavien–Dindo Grade I–V) occurred in 106 patients (49%), anastomotic leak occurred in 23 patients (11%), and there were 2 deaths (1%) within 30 days of surgery. The median length of hospital stay was 7 days (IQR 5–11). The median length of postoperative follow up was 27 months (IQR 16–46).

CT‐derived body composition parameters and CPET variables are shown in Table 2. The median SMI in men was 48.2 (IQR 42.3–53.5) cm^2^/m^2^, while in female, it was 39.9 (IQR 35.9–45.0) cm^2^/m^2^. The median SM‐MA in men was 38.8 (IQR 33.3–43.0) HU, and in female, it was 38.3 (IQR 32.0–44.8) HU. The median VO2 at AT was 12.2 (IQR 10.6–14.2) mL/kg/min, and the median VO2 peak was 18.8 (IQR 15.4–23.0) mL/kg/min. Body composition phenotypes are shown in Table 3. The prevalence of sarcopenia was 65% in men and 57% in female. Myosteatosis was present in 34% in men and 33% in female. Visceral obesity was present in 63% in men and 66% in female.

**Table 2:** Computed tomography derived body composition and cardiopulmonary exercise testing variables

	Male	Female	*P*‐value*
	Median [IQR]	Median [IQR]	
Skeletal muscle index (cm^2^/m^2^)	48.2 [42.3–53.5]	39.9 [35.9–45.0]	<0.001
Skeletal muscle mean attenuation (HU)	38.8 [33.3–43.0]	38.3 [32.0–44.8]	0.91
Visceral adipose tissue (cm^2^)	199.5 [129.5–273.6]	107.9 [53.9–176.9]	<0.001
Subcutaneous adipose tissue (cm^2^)	159.4 [114.7–231.9]	207.9 [146.1–293.4]	<0.001
VO2 at AT (mL/kg/min)	12.2 [10.6–14.2]		
	12.6 [11.1–15.1]	11.1 [9.5–12.8]	<0.001
VO2 peak (mL/kg/min)	18.8 [15.4–23.0]		
	20.1 [16.5–23.9]	16.4 [14.0–19.5]	<0.001

* Mann–Whitney *U* test.

**Table 3:** Body composition phenotype

	Male	%	Female	%
Sarcopenia				
No	54	35	27	43
Yes	99	65	36	57
Myosteatosis				
No	100	66	43	67
Yes	52	34	21	33
Visceral obesity				
No	56	37	22	34
Yes	97	63	43	66

The relationship between clinico‐pathological characteristics, body composition, and CPET variables are shown in Tables 4 and 5. On univariate linear regression analysis, male (*P* < 0.001) was positively associated with VO2 at AT, whereas ASA grade (*P* < 0.001) and BMI (*P* = 0.007) were negatively associated with VO2 at AT. On multivariate linear regression analysis, these variables remained statistically significant (*P* < 0.05).

**Table 4:** Relationship between clinico‐pathological characteristics, body composition phenotypes, and VO2 at AT in univariate and multivariate linear regression analysis

	Univariate			Multivariate		
	Beta	95% CI	*P*‐value	Beta	95% CI	*P*‐value
Age	−0.12	−0.08, 0.00	0.07	‐	‐	‐
Male	0.28	1.16, 3.07	<0.001	0.29	1.25, 3.11	<0.001
ASA (I–II, III–IV)	−0.22	−2.67, −0.70	<0.001	−0.20	−2.45, −0.58	0.002
BMI	−0.18	−0.24, −0.04	0.007	−0.18	−0.23, −0.05	0.004
T stage (0–2, 3–4)	−0.28	−1.16, 0.77	0.69	‐	‐	‐
N stage (0, 1–2)	−0.06	−1.32, 0.52	0.40	‐	‐	‐
Neoadjuvant treatment	−0.03	−1.52, 0.95	0.65	‐	‐	‐
Sarcopenia	−0.06	−1.38, 0.51	0.37	‐	‐	‐
Myosteatosis	−0.01	−1.02, 0.88	0.89	‐	‐	‐
Visceral obesity	−0.11	−1.74, 0.15	0.097	‐	‐	‐

**Table 5:** Relationship between clinico‐pathological characteristics, body composition phenotypes and VO2 peak in univariate and multivariate linear regression analysis

	Univariate			Multivariate		
	Beta	95% CI	*P*‐value	Beta	95% CI	*P*‐value
Age	−0.28	−0.22, −0.08	<0.001	−0.25	−0.21, −0.07	<0.001
Male	0.30	2.09, 5.28	<0.001	0.33	2.61, 5.50	<0.001
ASA (I–II, III–IV)	−0.23	−4.57, −1.28	<0.001	−0.14	−3.29, −0.21	<0.001
BMI	−0.20	−0.42, −0.09	0.003	−0.27	−0.49, −0.19	<0.001
T stage (0–2, 3–4)	−0.04	−2.10, 1.13	0.55	‐	‐	‐
N stage (0, 1–2)	−0.02	−1.38, 1.71	0.83	‐	‐	‐
Neoadjuvant treatment	−0.07	−3.15, 0.88	0.27	‐	‐	‐
Sarcopenia	−0.17	−3.50, −0.38	0.015	−0.17	−3.41, −0.59	0.006
Myosteatosis	−0.23	−4.28, −1.14	<0.001	−0.05	−2.16, 0.87	0.402
Visceral obesity	−0.12	−3.01, 0.15	0.075	‐	‐	‐

On univariate linear regression analysis, male sex (*P* < 0.001) was positively associated with VO2 peak. While age (*P* < 0.001), ASA grade (*P* < 0.001), BMI (*P* = 0.003), sarcopenia (*P* = 0.015), and myosteatosis (*P* < 0.001) were negatively associated with VO2 peak. On multivariate linear regression analysis age (*P* < 0.001), ASA grade (*P* < 0.001), BMI (*P* < 0.001), and sarcopenia (*P* = 0.006) were independently and negatively associated with VO2 peak.

## Discussion

Multiple studies have demonstrated that body composition is independently associated with outcomes following CRC surgery.^20^ Our data demonstrate the novel finding that sarcopenia is also independently associated with reduced VO2 peak on CPET in stage I–III CRC patients. In this study, sarcopenia is defined by sex and BMI specific threshold values that are known to predict poorer survival in CRC.^20^ Using this clinically relevant definition of sarcopenia, there appears to be a clear relationship between reduced muscle mass and function. Our findings support the notion that CT‐derived sarcopenia is a useful tool for recognizing poor preoperative fitness prior to CRC surgery. Although larger multicentred, prospective studies are needed to validate our finding, CT‐derived sarcopenia alone may be used as radiological biomarker of poor physical fitness without patients having to undergo the rigorous demands of CPET. Importantly, sarcopenia as defined in this study could be an alternative objective assessment metric for patients who are unable to undergo CPET due to limitations or contraindications.

Patients undergoing oesophago‐gastric cancer surgery had a pectoralis muscle mass that was positively associated with VO2 peak, supporting a relationship between muscle mass and physical fitness.^19^ In non‐malignant conditions, such as COPD, skeletal muscle loss, muscular wasting, and physical function impairment are twice as common.^25^ There appears to be significant decrease in VO2 peak and VO2 at AT in patients with a greater severity of COPD.^26^ Therefore, the relationship between thoracic body composition and physical fitness offers an interesting area meriting further investigation.

A previous study has demonstrated that sarcopenia is associated with reduced exercise capacity measured using VO2max and lower skeletal muscle capillarization.^27^ The participants were healthy volunteers without cancer, and sarcopenia was defined using appendicular lean mass derived from dual energy X‐ray absorptiometry divided by BMI. VO2max is defined as the metabolic rate at which oxygen uptake plateaus despite further increase in work rate.^3^ However, not all patients will achieve this end point during CPET limiting its clinical usefulness.^3^ Nonetheless, the findings suggest that reduced muscle capillarization may limit oxygen delivery and lead to chronic or intermittent hypoxia with muscle atrophy.^27^ Animal studies have shown that endothelial apoptosis and impairment in capillary function occurs prior to decline in muscle mass.^28^, ^29^ Endothelial dysfunction, insulin resistance, redox dysfunction, and oxidative stress are associated with decreased glucose uptake and mitochondrial dysfunction, leading to poor muscle function and fitness.^30^, ^31^, ^32^, ^33^

In clinical practice during the preoperative phase CRC patients identified as CT‐derived sarcopenia could be recruited for prehabilitation programme. Recognizing these high‐risk CRC patients would allow personalized discussion regarding the risk of surgery, enhance informed decision making, and consent process. During perioperative phase, sarcopenic CRC patients may benefit from being operated by senior experienced surgeon to optimize the risks of surgery. While in postoperative phase, these patients would benefit from critical care admission and close monitoring for complications. Therefore, we believe it is essential to recognize sarcopenic patients prior to CRC surgery.

In this study, sarcopenia is not associated with VO2 at AT. However, VO2 peak is an important CPET variable, and it is associated with higher complication rates and increased length of hospital stay as reported in a meta‐analysis with 1418 patients who underwent CRC surgery.^34^ Another meta‐analysis including 10 030 heterogeneous cancer patients, it was revealed that a higher VO2 peak was associated with improved post‐operative outcome.^2^ There is well‐established evidence to support the importance of VO2 peak in larger studies; therefore, we did not report on the clinical outcomes of our study cohort. The relationship between sarcopenia and VO2 peak remains an important finding despite, and no observable relationship with VO2 at AT in this study. A larger sample size may have confirmed such associations.

Myosteatosis is negatively associated with VO2 peak on univariate analysis, but not on multivariate analysis. There was no significant association between myosteatosis and VO2 at AT. Earlier work has demonstrated an association between myosteatosis and CPET‐derived VO2 at AT and VO2 peak in patients undergoing hepatopancreatobiliary surgery, which included metastatic disease.^18^ Another study has demonstrated myosteatosis is associated with metastatic disease in CRC.^35^ By excluding CRC patients with metastatic disease in our study, it is challenging to compare our findings with these previous studies. Nonetheless, it suggests that host body composition phenotype may alter as the disease progresses to a metastatic stage.

Visceral obesity was not associated with VO2 at AT or VO2 peak. Visceral obesity potentially increases cancer risk through adverse effects upon metabolism and inflammation.^36^ Visceral adipose tissue has endocrine, metabolic, and immunological function. Therefore, visceral obesity remains an important body composition phenotype in cancer. On the other hand, the widely used BMI criteria for overweight and obesity is unsuitable for determining visceral obesity. A large systematic review noted that BMI lacked sensitivity in identifying adiposity.^37^ However, in this study, BMI is negatively associated with VO2 at AT and VO2 peak in multivariate analysis. Our study suggests that despite BMI being a crude measurement of body composition, it remains an important surrogate of poor physical fitness.

Overall, the body composition phenotypes described in this study do not appear isolated in CRC patients. A large CRC cohort study demonstrated that a combination of sarcopenia and myosteatosis was present in 16% patients, whereas an amalgamation of sarcopenia, myosteatosis, and visceral obesity was present in only 4% of patients.^38^ This study concluded that multidimensional body habitus is independently associated with length of stay and hospital readmission, highlighting the importance of assessing the various body composition phenotypes. Due to our sample size, we were not able to analyse the relationship between different combinations of body composition phenotypes and CPET performance in our CRC cohort, and larger studies should be used to validate this study's findings and explore others.

Physical fitness prior to surgery is a modifiable risk factor.^39^ Poor physical fitness objectively assessed with CPET is associated with postoperative complications and increased length of hospital stay in CRC surgery.^34^ Prehabilitation is an evolving concept that aims to improve the functional capacity of deconditioned patients before major surgery. This is particularly relevant in the context of rectal cancer surgery in patients that receive neoadjuvant treatment; a previous study has demonstrated that neoadjuvant therapy is associated with significant decline in physical fitness in rectal cancer.^40^ Hence, the ability to improve functional capacity preoperatively may result in improved recovery overall after surgery. Yet the delivery of exercise interventions at home and in the community remains a challenge; not all patients may respond to or comply with such intervention; therefore, understanding the underlying pathophysiology of various body composition phenotypes such as sarcopenia, myosteatosis, and visceral obesity and their relationship with physical fitness needs greater clarity. The goal would be personalized cancer treatment pathway including host factors such as body composition in combination with tumour factors. Ultimately, the implementation of such approach would truly personalize CRC patient care.

This study represents a large CRC patient cohort assessed using CPET across multiple hospital sites that supports both the acceptability and the external validity of our findings. To our knowledge, this is the first study that has reported the relationship between sarcopenia defined by sex and BMI specific threshold values in CRC and objectively assessed fitness using CPET. By using prospective data collection, we have ensured low attrition of data points and avoided recall bias.

### Limitations

One institution in this study selectively used CPET for rectal cancer surgery. This may be due to the perceived higher risk of rectal cancer surgery when compared with colon cancer. The consequence from this approach has resulted in a higher proportion of male patients. The male patients achieved statistically higher median VO2 at AT and VO2 peak when compared with female in this study potentially introducing selection bias. The Frimley Park Hospital had implemented CPET service before the Whipps Cross University Hospital. The higher representation of patients from the Frimley Park Hospital may have introduced further bias.

## Conclusion

Sarcopenia is associated with poor physical fitness when objectively assessed using CPET in CRC patients undergoing curative surgery. Sarcopenia, yet again, proves to be an important clinical biomarker in CRC. Body composition and CPET results can be used in combination for risk stratification. Further work should be undertaken to assess whether CT‐derived body composition alone can perform as a suitable surrogate for CPET potentially enhancing a personalized approach to care.

## Conflict of interest

None declared.
